# Overview of anti-inflammatory diets and their promising effects on non-communicable diseases

**DOI:** 10.1017/S0007114524001405

**Published:** 2024-10-14

**Authors:** Xiaoping Yu, Haomou Pu, Margaret Voss

**Affiliations:** 1 School of Medicine and Nursing, Chengdu University, Chengdu 610106, People’s Republic of China; 2 School of Public Health, Chengdu University of Traditional Chinese Medicine, Chengdu 611137, People’s Republic of China; 3 Department of Nutrition and Food Studies, Falk College, Syracuse University, Syracuse, NY 13244, USA

**Keywords:** Inflammation, Anti-inflammatory diet, Non-communicable disease, Nutrition intervention, Mediterranean diet, Functional food, Nutrition therapy

## Abstract

An anti-inflammatory diet is characterised by incorporating foods with potential anti-inflammatory properties, including fruits, vegetables, whole grains, nuts, legumes, spices, herbs and plant-based protein. Concurrently, pro-inflammatory red and processed meat, refined carbohydrates and saturated fats are limited. This article explores the effects of an anti-inflammatory diet on non-communicable diseases (NCD), concentrating on the underlying mechanisms that connect systemic chronic inflammation, dietary choices and disease outcomes. Chronic inflammation is a pivotal contributor to the initiation and progression of NCD. This review provides an overview of the intricate pathways through which chronic inflammation influences the pathogenesis of conditions including obesity, type II diabetes mellitus, CVD, autoinflammatory diseases, cancer and cognitive disorders. Through a comprehensive synthesis of existing research, we aim to identify some bioactive compounds present in foods deemed anti-inflammatory, explore their capacity to modulate inflammatory pathways and, consequently, to prevent or manage NCD. The findings demonstrated herein contribute to an understanding of the interplay between nutrition, inflammation and chronic diseases, paving a way for future dietary recommendations and research regarding preventive or therapeutic strategies.

## Graphical abstract

**Figure:**
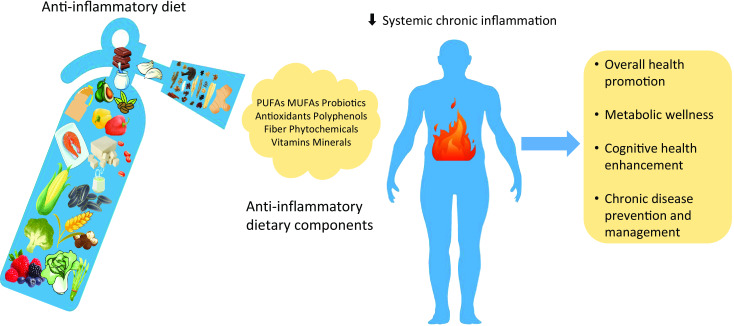

Chronic inflammation has become increasingly recognised as a significant contributor to the onset and progression of various non-communicable disease (NCD), acting through the persistent overproduction of pro-inflammatory agents such as oxidants, eicosanoids, cytokines and chemokines^(1)^. This sustained inflammatory condition can lead to tissue impairment and malfunction, barrier loss and infiltration of inflammatory cells, and it often results from a dysregulated immune response or prolonged exposure to irritants. These processes are further marked by elevated levels of high-sensitivity C-reactive protein (hs-CRP), IL-6 and TNF-α, which have also been correlated with major NCD^(2,3)^.

The mechanistic bridge that connects chronic inflammation to NCD is well-established and pivots on the detrimental effects of inflammation on cellular and systemic levels. For instance, in CVD, inflammation contributes to the formation of atherosclerotic plaques and is implicated in plaque instability and subsequent myocardial infarction^(4)^. In type 2 diabetes mellitus (T2DM), pro-inflammatory cytokines can induce insulin resistance by interfering with insulin signalling pathways^(5)^. Similarly, chronic inflammation is a recognised feature of cancer, where it facilitates tumour progression and metastasis^(6)^, and cognitive disorders have also been linked to systemic inflammation, which is believed to promote neuronal damage and cognitive decline^(7)^. Given the implications of chronic inflammation in NCD, there is increasing focus on dietary patterns and individual nutrients that may modulate inflammatory processes.

So far, anti-inflammatory diets have garnered attention as a lifestyle approach to modulating this unregulated immune response. They are characterised by high consumption of fruits, vegetables, whole grains, legumes, fatty fish, nuts, olive oil and phytochemicals^(8)^, while limiting the intake of foods with potentially pro-inflammatory properties such as red meat, refined carbohydrates and alcohol^(9,10)^. The rationale for anti-inflammatory diets (including but not limited to the Mediterranean diet (MD)) lies in their capacity to decrease levels of pro-inflammatory biomarkers and positively influence pathways involved in the inflammatory cascade.

Anti-inflammatory diets, therefore, serve a dual purpose: they provide nutrients that can directly dampen the inflammatory response, and they also modulate the body’s metabolic processes to fortify against NCD. Foods rich in *n*-3 fatty acids, antioxidants and polyphenols have been shown to reduce the expression of pro-inflammatory genes and decrease circulating levels of inflammatory biomarkers^(11,12)^. These dietary components have also been associated with improved endothelial function, reduced oxidative stress and enhanced insulin sensitivity, thereby lowering the risk and severity of diseases like CVD and T2DM^(13,14)^. In this paper, we review the current body of evidence regarding anti-inflammatory diets and their components to explore how dietary choices can influence inflammatory processes and impact the prevention and management of chronic diseases.

## Anti-Inflammatory diet components

Anti-inflammatory diets are distinguished by a strategic selection of foods rich in key nutrients including dietary fibre, vitamin C, vitamin E, ω-3 fatty acids, Zn and polyphenols^(15,16)^. Understanding the major components (Table 1) of this dietary approach is essential to evaluating its potential effect on chronic inflammation and, subsequently, chronic diseases. It is also worth noting that the anti-inflammatory properties of food components extend beyond their individual effects. In other words, some of them exhibit synergistic effects when consumed together, such as prebiotics (e.g. fibre) and probiotics (e.g. microbes). The combination of these two components has been shown to have a more profound anti-inflammatory impact than either component alone^(26)^. Vitamins C and vitamin E are potent antioxidants that can quench free radicals and, as a result, reduce oxidative stress, which is an underlying factor in inflammation^(27)^. Moreover, Zn contributes to the defense against oxidative damage by supporting cytosolic Zn/Cu superoxide dismutase, blocking NADPH oxidase and promoting cysteine-rich metallothionein production^(28)^. *n*-3 fatty acids also work synergistically with vitamin E^(29–31)^. This relationship may be due to the ability of vitamin E to decrease oxidative stress induced by these fatty acids, thus maintaining their anti-inflammatory function^(30)^. Lastly, the consumption of olive oil polyphenols is implicated in the anti-inflammatory benefits linked to the MD^(32)^, rich in *n*-3 fatty acids and a combination of *n*-3 fatty acids and phenolic compounds have potentially demonstrated anti-inflammatory effects^(33)^.

**Table 1.** Major components, sources and beneficial effects of an anti-inflammatory diet

Category	Source	Anti-inflammatory effect	Reference
ω-3 fatty acids	Fatty fish (salmon, mackerel), flaxseed, chia seeds, walnuts and fish oil supplements	Inhibit leukocyte chemotaxis, reduce the expression of adhesion molecules and interactions between leukocytes and endothelial cells, decrease the generation of eicosanoids derived from ω-6 fatty acids (e.g. arachidonic acid) and suppress inflammatory cytokines synthesis and T cells reactivity.	Calder^(17)^
MUFA	Olive oil, avocados, nuts and seeds (almonds, cashews, peanuts and sesame), canola oil	Increase adiponectin secretion, improve insulin resistance and reduce proinflammatory cytokines and circulating monocytes.	Ravaut *et al.* ^(18)^
Antioxidants	Fruits (berries, citrus, etc.), vegetables (spinach, kale, etc.), nuts, seeds, herbs (turmeric, ginger, etc.)	Alleviate oxidative stress, neutralize free radicals and protect against DNA damage brought on by hydroxyl radicals.	Griffiths *et al.* ^(19)^
Polyphenols	Extra virgin olive oil, black/green tea, nuts, seeds, dark chocolate, legumes, fruits, red wine, vegetables and coffee	Exhibit anti-inflammatory and antioxidant properties and have the potential to mitigate blood pressure, lipid profiles, abdominal obesity, blood glucose and vascular conditions.	Kiyimba *et al.* ^(20)^
Dietary fiber	Whole grains, fruits, vegetables, legumes, nuts and seeds	Increases anti-inflammatory short-chain fatty acids production in the colon, reduces proinflammatory cytokines and helps to maintain a healthy gut microbiome and a balance of proinflammatory and anti-inflammatory compounds.	Dürholz *et al.* ^(21)^
Phytochemicals	Turmeric (curcumin), ginger, garlic, cinnamon and other herbs and spices	Modulate inflammatory pathways, reduce proinflammatory cytokines and exert antioxidant activities.	Jiang *et al.* ^(22)^
Probiotics	Yogurt, kefir, sauerkraut, pickles and other fermented foods	Improve epithelial barrier function, reduce lipopolysaccharide (LPS)-dependent chronic low-grade inflammation and biomarkers associated with metabolism, inflammation and oxidative stress.	Hutchinson *et al.* ^(23)^
Vitamins and minerals	Citrus fruits, berries, vegetables, nuts, seeds, legumes and fish	Exert antioxidant properties; involved in anti-inflammatory processes.	Vahid *et al.* ^(24)^
Low glycaemic index (GI) foods	Whole grains, legumes, non-starchy vegetables	Regulate postprandial blood glucose levels, alleviate oxidative stress and reduce inflammatory markers.	Rondanelli *et al.* ^(25)^

However, it is equally important to acknowledge that not all food components work in harmony. In some cases, the presence of certain components can antagonise one another, leading to reduced absorption and bioavailability^(34)^. For example, phytate, a compound found in many plant-based foods, can inhibit the absorption of minerals such as Zn and Fe^(35)^. Therefore, it is necessary to consider these complex interactions between food components when evaluating their anti-inflammatory effects.

### Fruits and vegetables

Central to an anti-inflammatory diet are fruits and vegetables, especially those rich in polyphenolic compounds. For instance, red grapes and wine are sources of resveratrol, a non-flavonoid polyphenol that has been recognised for its anti-inflammatory and antioxidant properties^(36–38)^. Resveratrol modulates key inflammatory mediators, including cyclooxygenases (COX) and cytokines such as TNF-α, through the inhibition of the nuclear factor-kappa B (NF-κB) pathway, a pivotal molecular cascade in the inflammatory response^(39)^. COX enzymes also transform arachidonic acid to prostaglandins, and by inhibiting this process, inflammation is decreased^(39)^. Berries exhibit a rich profile of flavonoids, including anthocyanin, which have been observed to modulate the expression of inflammatory cytokines and play a role in NCD prevention and management^(40–43)^. Vegetables including onions, asparagus and broccoli, beyond their well-noted fibre and vitamin contents, are also a valuable source of flavonoids such as kaempferol and quercetin^(44)^. These nutrients possess anti-inflammatory properties by acting as antioxidants, modulating pro-inflammatory enzyme activities and gene expression and inhibiting transcription factors, including TNF-α, inducible nitric oxide synthase (iNOS), COX-2, IL-6, IL-8 and NF-κB^(45,45,46)^. Additionally, citrus fruits contribute to this anti-inflammatory arsenal also due to flavonoids such as hesperidin and naringenin, which have potentials in regulating oxidative stress and inflammatory pathways^(47)^.

In addition, flavonoids and their metabolites inhibit the production of reactive oxygen species and reactive nitrogen species in neutrophils and macrophages^(48)^. These compounds suppress the activity of enzymes involved in the generation of reactive oxygen species and reactive nitrogen species, including NADPH oxidase, myeloperoxidase and iNOS^(48)^. This suggests that flavonoids may play a role in modulating immune responses and potentially protect against inflammation and tissue damage. Moreover, anthocyanins, mainly presented in red to dark blue coloured fruits and vegetables, have been intensively studied for their antioxidant activities, suggesting their potential anti-inflammatory effects through scavenging radicals and alleviating oxidative stress^(49)^.

### Whole grains

Brown rice, barley, quinoa, oats, corn and millet retain the essential composition of their endosperm, germ and bran in consumed form. They not only serve as primary sources of carbohydrates but are also integral components of an anti-inflammatory diet. Specifically, whole grains provide a range of antioxidant vitamins (e.g. vitamin B_6_, thiamine, folate and vitamin E), as well as phytochemicals and dietary fibre that are precursors to important microbial-derived metabolites (e.g. phenolic acids and short-chain fatty acids)^(50)^. These collectively contribute to the regulation of inflammatory processes. Animal studies^(51,52)^ assessed the impact of whole grain intake on inflammatory biomarkers and showed reduced inflammation, as evidenced by decreased levels of IL-1β, NF-κB, TNF-α, LPS-binding protein, iNOS, IL-6 and monocyte chemoattractant protein-1 in rodent models.

The anti-inflammatory effects of whole grains in human studies remain controversial. While comparing the impact of consuming whole grains on inflammatory markers with that of consuming refined grains, a systematic review and meta-analysis which included thirteen randomised controlled trials (RCT) with 466 participants indicated a statistically significant decrease in hs-CRP concentrations and IL-6 levels^(53)^. However, there was no significant reduction in serum concentration of TNF-α observed following whole grain intake^(53)^. Similarly, Milesi *et al.* ^(54)^ concluded significant reductions in circulating inflammatory biomarkers, particularly CRP among both obese/overweight populations and those with pre-existing health conditions, and, across twelve out of thirty-one RCT examined, there existed significant associations between whole grain consumption and lower levels of at least one inflammatory marker among CRP, IL-6 and TNF. These results suggest an increase in whole grain consumption may be an effective strategy for modulating pro-inflammatory responses in the body, which could have implications for overall health and disease prevention.

### Nuts and seeds

The consumption of nuts and seeds is associated with improved lipid profiles, reduced risk of CVD and beneficial effects on lipid metabolism and oxidative stress^(55)^. Rich in MUFA and PUFA, soluble fibre, phenolic compounds, vitamins, and minerals, nuts and seeds have been shown to decrease serum total cholesterol, TAG and LDL-cholesterol levels^(56)^. Moreover, nuts may exert anti-inflammatory effects. Hong *et al.* ^(55)^ reported significant reductions in serum CRP and high mobility group box 1 protein in rats fed with pistachio and mixed nuts, while similar reductions in hs-CRP, adiponectin and TNF-α levels have been observed in humans consuming pistachios^(57)^. A systematic review and meta-analysis^(58)^ focused on almond consumption demonstrated significantly reduced serum concentrations of CRP and IL-6, particularly at doses below 60 g/day, but found no significant effects on TNF-α, intercellular adhesion molecule 1, or vascular cell adhesion molecule 1, with benefits being less pronounced in unhealthy or obese individuals. Another meta-analysis^(59)^ found nut consumption significantly reduced intercellular adhesion molecule levels, especially with mixed nuts and in long-term studies of 12 weeks or more, but had no significant impact on other inflammatory markers. These findings suggest that the effects may vary depending on the type of nut, duration of consumption and individual health conditions such as obesity.

It is also worth noting that seeds oils (e.g. sunflower seed, poppy seed and sesame seed) tend to have higher concentrations of ω-6 fatty acids, while flaxseed and hempseed oil are rich in ω-3 fatty acids^(60)^. Although ω-6 fatty acids (e.g. arachidonic acid) are essential, they are also a precursor to a number of pro-inflammatory mediators such as prostaglandins and leukotrienes^(61)^. However, a systematic review of RCT indicated that increased intake of arachidonic acid, up to 1000–1500 mg/d, in adult did not appear to have adverse effects on inflammation or immune function, but insufficient data were available to draw specific conclusions^(62)^. Additionally, nuts and seeds are energy-dense foods and could contribute to weight gain if consumed excessively.

Intriguingly, epidemiological studies^(63–65)^ have demonstrated inverse relationships between nut consumption and obesity. Systematic reviews and meta-analyses^(66–68)^ have also shown that nut consumption does not lead to weight gain and is associated with a decreased risk of overweight and obesity^(66)^. Specific nuts, such as almonds, have been linked to reductions in waist circumference, while walnuts are associated with a lower body fat percentage^(67)^. Therefore, with regard to nuts and seeds, tailored approaches considering individual factors such as allergies and energetic intakes are essential to maximise their benefits while minimising potential risks.

### Protein sources

The recommended protein sources for anti-inflammatory diets are primarily lean meats or plant based. While some animal proteins are present in an anti-inflammatory diet, the proteins derived from plant-based sources including legumes, nuts, soya, seeds and mushrooms are suggested. Recent research has found that peptides isolated from plant proteins, specifically those of the Leguminosae family such as soybeans and beans, can modulate some pro-inflammatory molecules (e.g. eicosanoids, prostaglandin E2 (PGE2), nitric oxide (NO), iNOS, COX-2, as well as many cytokines/chemokines)^(69)^. Of particular interest are three peptides: lunasin, VPY and γ-glutamyl peptides, which have demonstrated potent anti-inflammatory properties^(70–72)^. Lunasin inhibits the activation of NF-κB pathway and reduces the transcription of cytokines IL-1β and IL-6, as well as enzymes such as COX-2 and iNOS, which are instrumental in the synthesis of eicosanoids and NO^(73,74)^. Soybean tripeptide VPY has been observed to lower MPO activity and decrease expressions of TNF-α, IL-1β, IL-6, IL-17 and interferon-γ (IFN-γ) through PepT1 transporters^(71)^. Additionally, γ-glutamyl peptides may be associated with the downregulation of the c-Jun N-terminal Kinase and NF-κB signalling pathways via calcium-sensing receptor (CaSR), influencing the production of inflammatory mediators^(75)^.

Furthermore, the consumption of soya products has been linked to reduced levels of inflammatory markers, including IL-6, TNF-α and CRP^(76,77)^. The anti-inflammatory effects of soya are mediated by its phytoestrogen content, particularly daidzein and genistein, which have been shown to reduce systemic inflammation^(77)^. Genistein inhibits NF-κB activation by blocking NF-κB translocation to the nucleus^(78)^, and daidzein can reduce the synthesis of NO and IL-6, along with their mRNA expression, while also decreasing reactive oxygen species production, p38 MAPK phosphorylation and NF-κB activation^(79)^. In addition, both daidzein and genistein exhibit anti-inflammatory effects by decreasing the release of IL-6, IL-8 and monocyte chemoattractant protein-1 induced by lipopolysaccharide^(80)^.

In animal products, on the other hand, the ratio of essential fatty acids contributes to the overall effect on inflammation^(81)^. For instance, fatty fish, a source of ω-3 fatty acids, has well-documented anti-inflammatory properties, while excessive consumption of red or processed meats, high in ω-6 and saturated fatty acids, may tip the balance towards a more pro-inflammatory state^(82)^. In general, diets high in animal-based proteins are correlated with increased levels of inflammatory markers, potentially due to the presence of pro-inflammatory compounds, such as heme iron^(83)^ and advanced glycation end products when cooked at dry and high temperatures^(84)^.

### Fermented foods

Traditionally, fermentation has been employed as a preservation technique for foods, but this process has gained recent attention due to its ability to enhance nutritional qualities and produce bioactive compounds with promising health benefits^(85)^. Current studies have demonstrated the beneficial effects of fermented foods on several health issues, suggesting that they may offer protection against diabetes, cancers and inflammatory disorders by virtue of their content of probiotics, peptides, polyphenols, short-chain fatty acids and other bioactive compounds^(85–87)^. For example, fermented blueberries and blackberries contain high concentrations of polyphenolic compounds with enhanced bioavailability. These compounds regulate key inflammatory signaling pathways including MAPK/ERK, STAT3, NF-κB and PI3K/AKT, and they exhibit potent antioxidant capabilities, potentially aiding in the reduction of chronic inflammation and LDL oxidation^(88–91)^.

Equally, fermented products owe their potential health benefits to the active participation of beneficial microorganisms^(92)^. These microbial constituents not only contribute to maintaining a balanced gut microbiota but also regulate inflammatory processes and gut barrier integrity, thereby protecting against various diseases^(93–96)^. Commensal bacteria contribute to the structural integrity of the gut barrier. They stimulate the production of tight junction proteins, which prevent the translocation of pathogenic bacteria and toxins into the host circulatory system, thus reducing inflammatory responses^(97)^. Short-chain fatty acids, including butyrate, propionate and acetate, are produced via the fermentation of dietary fibres by gut microbiota^(98)^. Short-chain fatty acids, particularly butyrate, modulate the inflammatory response by suppressing NF-κB activation and TNF-α, IL-6 and NO secretion stimulated by lipopolysaccharide and cytokines^(99,100)^.

In fermented dairy products such as yogurt, kefir and cheese, proteins are denatured during fermentation, resulting in the liberation of short peptide fragments with potential health benefits. Among these peptides, angiotensin-converting enzyme (ACE) inhibitors have garnered particular interest due to their ability to exhibit anti-hypertensive effects involving the sequestration of ACE by the C-terminal sequence of ACE-inhibitors, which hinders ACE’s ability to convert angiotensin I into angiotensin II^(101)^. Furthermore, fermented milked products have been shown to possess anti-inflammatory properties. In clinical trials, Wang *et al.* ^(102)^ observed increased levels of lipopolysaccharide-binding protein and IL-10 in participants who received fermented milk enriched with *Lactobacillus casei* Zhang and *Bifidobacterium animalis* ssp. lactis V9 (PFM) for 4 weeks. Participants also exhibited lower concentrations of CRP, suggesting that the consumption of PFM may lead to a reduction in systemic inflammation through various mechanisms involving lipopolysaccharide-binding protein and IL-10^(102)^. In addition, lactic acid bacteria and *Bifidobacterium* strains present in fermented dairy products have also been reported to suppress inflammation by modulating the immune response, reducing oxidative stress and inhibiting the production of pro-inflammatory cytokines^(103,104)^. These mechanisms are thought to underlie their ability to mitigate inflammation in a variety of contexts, including gastrointestinal disorders, allergies and autoimmune diseases.

### Spices and herbs

Anti-inflammatory diets often incorporate various spices and herbs known for their bioactive compounds with anti-inflammatory and antioxidant properties^(48)^. Some studies suggest that the use of culinary spices and herbs exhibit anti-inflammatory properties by activating PPAR α and γ (PPAR γ)^(105)^. PPAR γ activators are capable of suppressing NF-κB activation, leading to the downregulation of pro-inflammatory cytokines. Consequently, the spices in our diet may function as broad-spectrum PPAR activators that can improve insulin sensitivity, mitigate dyslipidaemia and counteract weight gain^(105)^. These include apigenin (found in marjoram, sage, thyme and holy basil)^(106)^, capsaicin (in chili pepper)^(107)^, curcumin (in curcuma)^(108,109)^, eugenol (in clove)^(110)^ and gingerol and 6-shogaols (in ginger)^(111,112)^, serving to attenuate the harmful effects of chronic inflammation induced by obesity, consequently retarding the progression of diseases associated with prolonged inflammatory responses.

Garlic, ginger, rosemary, peppers, clove, basil, oregano, cumin, turmeric, cinnamon and thyme are among the most frequently used spices and herbs. A wide range of health benefits have been reported for these plant products due to rich concentrations of bioactive flavonoids, polyphenols, tannins, alkaloids, sulphur-containing compounds and other constituents^(22,113)^. Possible benefits include protection against CVD, neurodegenerative diseases, metabolic syndrome and cancer^(105,114–116)^. In the context of chronic diseases and inflammation, Kunnumakkara *et al.* ^(117)^ elucidated the mechanisms underlying some bioactive constituents of spices, including α-Pinene, 1,8-cineole, 6-gingerol, allicin, anethole, capsaicin, curcumin, quercetin, eugenol and others. These compounds are found in rosemary, basil, ginger, garlic, anise, red pepper, turmeric, onions and clove. These components exhibit efficacy against chronic diseases through the inhibition of NF-κB, STAT3 and ERK/MAPK pathways, accompanied by a reduction in inflammatory cytokines TNF-α, IL-1, -2, -4, -6, -8 and chemokines. However, there is a paucity of human interventions assessing the impacts of these compounds in their natural, whole-food state^(89)^. Further investigation is needed to evaluate their absorption, bioavailability and potential synergistic or antagonistic effects when ingested in whole foods or in conjunction with other foods. Even so, given the adverse effects and escalating expenses associated with contemporary therapeutics, spices and herbs and their bioactive constituents present huge potential for the formulation of cost-effective, innovative and secure treatments targeting chronic diseases. Table 2 shows examples of phytochemical compounds commonly found in spices and herbs as well as the range of mechanisms by which they may exert anti-inflammatory effects.

**Table 2.** Phytochemical compounds in spices and herbs and their possible anti-inflammatory effects—source information: Bellik *et al.* ^(118)^, Jungbauer *et al.* ^(105)^, Daly *et al.* ^(119)^, Ganeshpurkar *et al.* ^(120)^, Dixon *et al.* ^(121)^, Baur *et al.* ^(122)^, Calderón-Montaño *et al.* ^(123)^, Isemura^(124)^. Checkmarks indicate the presence of a compound for each activity

Phytochemical compounds	Enhance antioxidant activities	Protection against free radicals	Suppress cellular communication in immune cells	Inhibit arachidonic acid metabolism	Inhibit pro-inflammatory enzymes (e.g. COX, LOX, NOS)	Inhibit NF-κB activation and inflammation-related proteins/enzymes	Dietary sources
β-carotene	☑	☑				☑	Basil, coriander
Rutin		☑		☑		☑	Passionflower, buckwheat
Genistein			☑			☑	Soybean seeds
Resveratrol	☑				☑	☑	Grapes, wine, Itadori tea, Rhubarb dry root
Quercetin	☑	☑	☑	☑	☑	☑	Dill, bay leaves, oregano
Curcumin	☑	☑			☑	☑	Turmeric
Kaempferol			☑	☑	☑	☑	Oregano, endive, leek, Ginkgo biloba
Luteolin	☑	☑	☑		☑	☑	Marjoram, sage, rosemary, tarragon, thyme
Catechin	☑				☑		Green tea, apples, persimmons, cacaos

### Dietary inflammatory index

The Dietary inflammatory index (DII) was first published in 2009 to assess the inflammatory impact of individuals’ diets and to quantify the inflammatory properties of foods and nutrients by considering their effects on CRP^(125)^. Thorough validation procedures have then been applied to the DII to ensure its reliability across various demographic groups, and its development was rooted in a comprehensive examination and synthesis of scientific literature regarding the relationship between diet and inflammation^(126)^. After that, subsequent validation efforts have confirmed its ability to effectively evaluate the inflammatory impact of diets and its consistent performance across diverse population^(127–136)^. These endeavours encompassed both observational investigations and clinical trials, underscoring the DII’s resilience in gauging dietary inflammatory potential.

The DII calculation focuses on nutrients and foods which can affect the body’s inflammatory biomarkers, including TNF-α, CRP, IL-6, IL-1β, IL-4 and IL-10. For example, pro-inflammatory components include saturated fat, trans fat, cholesterol, Fe, Na, processed meats, alcohol and sugary beverages, while ω-3 fatty acids, fibre, vitamins (e.g. A, C, E and D) and minerals (e.g. Mg and Zn) are considered anti-inflammatory^(126)^. Moreover, anti-inflammatory dietary patterns such as the MD are associated with reduced inflammatory status^(137)^. A greater DII score suggests a diet with increased pro-inflammatory potential, while a lower DII score signifies a diet with greater anti-inflammatory properties. Studies have shown that diets with higher DII scores are linked to elevated risks of NCD such as CVD, cancer, metabolic diseases and cognitive disorders, but the evidence supporting most outcomes remains inconclusive, highlighting the need for further prospective studies to refine these associations^(138)^.

## Anti-inflammatory diet and non-communicable diseases

Inflammation is the body’s immune response to injury, infection or harmful stimuli^(117)^. This complex biological reaction aims to mitigate potential sources of cellular and tissue damage by invoking the immune system to safeguard the body and facilitate recovery. At its core, inflammation entails triggering the immune system to defend against pathogens, removing contaminated cells and tissues and initiating tissue restoration through a carefully orchestrated sequence of events^(139)^. These events include vascular dilation, increased permeability of blood vessels, attachment of cells to the injury site, immune cell migration, molecular mediator release and tissue repair^(140)^. While acute inflammation represents a necessary and local defense mechanism, it is important to recognise that uncontrolled or chronic inflammation can arise from immunological imbalances, leading to detrimental consequences for overall health.

**Fig. 1.**
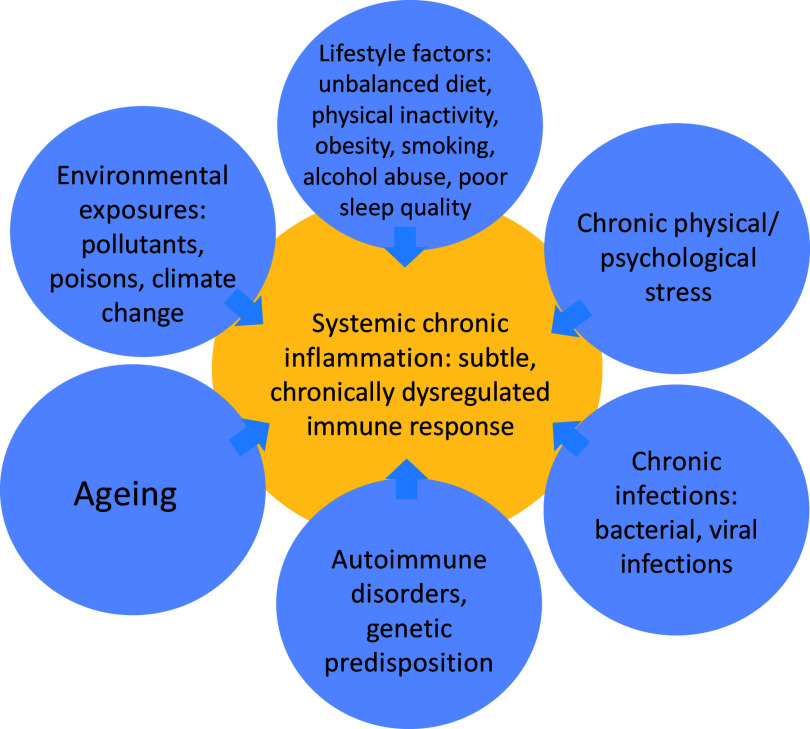
Common causes of systemic chronic inflammation.

A wealth of existing research underscores the intricate and pervasive link between chronic inflammation and major NCD^(141,142)^. Figure 1 identifies potential contributors to chronic inflammation. Chronic inflammation, along with mitochondrial alterations and oxidative stress, serves as common denominators in the pathogenesis and progression of a spectrum of NCD, such as obesity, CVD, diabetes and certain cancers^(143,144)^. Employing therapeutic interventions to mitigate oxidative stress and inflammation holds great promise for reducing mortality and morbidity rates associated with various diseases. As such, it also offers avenues for targeted dietary interventions to cope with this inflammatory burden and improve overall health outcomes.

### Obesity

Obesity is a complex medical condition characterised by excess body fat and high levels of BMI. This condition has been shown to be closely linked to the development of insulin resistance, a precursor to T2DM^(145)^. Moreover, imbalances in adipokines released by fat tissue contribute to inflammation-related metabolic issues^(146)^, and obese individuals are at a higher risk of acquiring dyslipidaemia, hypertension, T2DM and CVD^(147–150)^. Research evidence supports a link between obesity and an elevated inflammatory state^(151)^. As the amount of subcutaneous and/or abdominal fat increases, it leads to adipose tissue inflammation which involves a range of complex changes within the adipose tissue itself, including lipid storage, adipocyte hypertrophy and enhanced cell death. Additionally, adipose tissue serves as both a primary fat storage site and a significant endocrine organ, releasing adipokines and cytokines that can influence physiological and pathological processes throughout the body, such as leptin, adiponectin, resistin, visfatin, omentin and chemerin^(152,153)^. Within the adipose tissue, M2-like adipose tissue macrophages play important roles in maintaining tissue homeostasis through suppressing adipocyte precursor proliferation, promoting dead adipocyte removal and releasing anti-inflammatory cytokines like IL-1Rα, IL-4, IL-10 and IL-13. However, when adipose tissue function becomes impaired due to obesity, adipocytes start secreting pro-inflammatory cytokines, including IL-6, IL-8, monocyte chemoattractant protein-1 and TNF-α, attracting more circulating monocytes and immune cells into the adipose tissue, exacerbating the inflammatory environment^(154)^.

The development and progression of adipose tissue inflammation can be influenced by obesity-related factors, including dietary patterns, genetic predisposition and gut microbiome composition^(155)^. In this context, an anti-inflammatory diet may serve as a valuable strategy for weight loss and managing inflammation within adipose tissues. Numerous studies have documented the positive effects of an anti-inflammatory dietary intervention on weight loss, highlighting the significance of consuming ample dietary fibre, mono- and polyunsaturated fatty acids, plant-based and lean protein, polyphenols and probiotics in achieving this outcome^(156–160)^.

A four-month cross-sectional study of 535 adolescent participants^(157)^ reported that individuals with a lower DII tended to consume more fibre, lutein and beta-carotene, while simultaneously consuming less SFA, MUFA and PUFA, as well as oleic acid and linoleic acid. The study also discovered that a higher body fat percentage was positively correlated with DII levels (odds ratio (OR: 1·6, 95 % CI: 1·1, 2·3, *P* = 0·019)^(157)^. In an adult population^(159)^, over the course of a 6-month randomised controlled trial, participants following an energy-restricted anti-inflammatory diet showed significant improvements in body weight, visceral fat and measures of cardiovascular risk factors, including waist circumference, BMI and HDL cholesterol levels. Additionally, there was evidence of improved inflammation markers hs-CRP, IL-6 and TNF-α concentrations^(159)^. Lastly, Lotfi *et al.* ^(160)^ uncovered a substantial negative correlation between adherence to the MD and odds of developing obesity or becoming overweight. Specifically, they discovered that individuals who closely followed the MD were 9 % less likely to experience excess weight (OR: 0·91; 95 % CI: 0·88, 0·94; interaction test probability (PQ-test): 0·031). The association remained statistically significant when analysing data from studies examining both overweight and obesity outcomes together (OR: 0·92; 95 % CI: 0·88, 0·96; PQ-test: 0·166), yet lost significance when evaluating only obesity outcomes (OR: 0·68; 95 % CI: 0·43, 1·10; PQ-test: 0·132). Additionally, linear dose–response modelling of six studies indicated a 2 % lower risk of overweight or obesity for every one-point increase in the MD score (OR: 0·98; 95 % CI: 0·96, 0·99)^(160)^. Furthermore, each increment in the MD score was found to be correlated with a slight reduction in weight gain over 5 years (–0·04 kg; 95 % CI: –0·07, –0·02 kg; *n* 13 from four cohorts)^(160)^. In general, adherence to diets with anti-inflammatory properties, characterised by a low glycaemic index and high levels of fruits, legumes, vegetables, nuts, whole grains, fish, lean meat and healthy fats, while minimising processed foods, added sugars, saturated or trans-fatty acids, red meat, refined grains and sodium intake, can lead to significant reductions in body weight and BMI^(158)^. These findings are thought to be mediated by several mechanisms, including improved insulin sensitivity, enhanced fat metabolism and reduced inflammation and oxidative stress, all of which contribute to a more favourable energy balance and increased fat oxidation.

### Type 2 diabetes mellitus

The rising prevalence of diabetes is largely attributed to unhealthy behaviours and increasing rates of obesity^(161)^. As mentioned earlier, excess body weight is a precursor to T2DM, and inflammatory pathways are proposed as common underlying mechanisms linking obesity, T2DM and CVD. Chronic inflammation, with many inflammatory biomarkers originated from adipocytes, is consistently observed in diabetic patients, correlating with the prevalence, incidence and severity of diabetes and its complications^(162–166)^. One possible mechanism involves persistently elevated levels of leptin produced by fat cells in obese individuals^(167)^. Higher leptin secretion has been linked to increased production of pro-inflammatory cytokines, such as TNF-α, IL-1β and IL-6^(168)^. These cytokines, along with hs-CRP, have been implicated in the pathogenesis of insulin resistance and the development of chronic diseases such as T2DM^(169–171)^. At present, there is no known cure for diabetes, and the most effective approach to preventing the disease is through lifestyle modifications that involve following a healthy diet, weight reduction and increase in physical activity, with estimates suggesting a 40–70 % of decrease in risk^(172)^.

Some evidence suggests that diets can lead to beneficial changes in circulating inflammatory biomarkers, while systemic chronic inflammation may contribute to the pathophysiology of T2DM^(162,173)^. Adopting anti-inflammatory dietary patterns appears to play a role in its prevention and management. For example, a cross-sectional analysis of 4434 adult participants discovered an association between DII scores and the risk of developing diabetes, with each increase in the DII score corresponding to a 13 % increase in the odds of diabetes (95 % CI: 1·02, 1·24)^(174)^. The severity of diabetes was also significantly associated with higher DII scores. Furthermore, a 43 % increase in the odds of having HbA1c levels above 9 % was observed for every 1-point increase in the DII score (95 % CI: 1·21, 1·68)^(174)^. Notably, this association was stronger among individuals with HbA1c levels above 9 %. Similar results were reported by Laouali *et al.* ^(175)^. However, this study found that a diet rich in anti-inflammatory properties was inversely associated with the risk of developing T2DM, with the potential mediating role of BMI^(175)^.

Despite the relationships outlined above, some studies have not yielded consistent associations^(176,177)^, and a recent meta-analysis involving 1 687 424 participants revealed no significant correlation between DII and the risk of T2DM^(178)^. A randomised controlled feeding study of diabetic and pre-diabetic patients, investigating the effects of an anti-inflammatory diet compared with a control diet based on American Diabetes Association recommendations on body weight and inflammation markers, could not identify a specific advantage to eliminating foods commonly linked to inflammation^(179)^. Any improvements in inflammation may be due to weight loss rather than the specific elimination of inflammatory foods or the addition of anti-inflammatory fats^(179)^. Another systematic review and meta-analysis of RCT observed a decrease in CRP and an increase in adiponectin in diabetic adults following the MD, Diabetes Prevention Program, and Diabetes UK healthy eating^(162)^. Al-Aubaidy and colleagues noted a rise in plasma citrus bioflavonoids levels along with a decrease in IL-6 among T2DM patients after 12 weeks of adhering to the MD^(180)^, highlighting the potential impact of polyphenol-rich citrus fruits. Additionally, the inclusion of raspberries and resveratrol supplements led to improved glucose homeostasis and reductions in serum inflammatory markers^(37,40)^. These findings may implicate that an anti-inflammatory dietary pattern plus supplementation with polyphenol-rich foods may enhance anti-inflammatory effects and generate more favourable health outcomes in patients with T2DM.

In participants with pre-diabetes and insulin resistance, on the other hand, DII exhibited a positive correlation with fasting plasma glucose, fasting serum insulin and the homeostatic model assessment of insulin resistance, suggesting that a diet with higher inflammatory potential was linked to elevated odds of insulin resistance and prediabetes^(181)^. We also found that individual anti-inflammatory food components such as dietary fibre, ω-3 fatty acids, curcumin, anthocyanin and other polyphenols, either alone or in combination, may improve diabetic outcomes and reduce levels of inflammatory markers^(40,182–185)^. However, studies exploring holistic dietary patterns have yielded inconsistent findings regarding the association between dietary inflammatory potentials and the incidence of T2DM.

A question arises as to whether the dietary pattern that promotes weight loss also reduces inflammation and diabetic risk, or if an anti-inflammatory dietary pattern independently contributes to these outcomes? Unfortunately, there is currently minimal research to support any conclusion. Further studies are needed to confirm this relationship.

### Cardiovascular disease

CVD (e.g. CHD, heart failure and CVD, etc.) is the foremost cause of mortality globally^(186)^. Chronic inflammation may contribute to the development of CVD^(187–189)^. In prospective cohort studies, elevated levels of inflammatory biomarkers, tumour necrosis factor-α receptor 2 (TNFα-R2), soluble intercellular adhesion molecule-1, CRP and IL-6, have all been associated with an increased risk of CVD^(190)^. Additionally, randomised trials have also provided evidence for the causal link between inflammatory cytokines and CVD pathogenesis, such as IL-1β and IL-6^(191,192)^. Various factors, such as gender, smoking, medication, physical activity level, age and diet, play a role in the inflammatory process^(193)^. Therefore, diet, as an adjustable exposure, has been linked to the aetiology of CVD through its influence on inflammation, and several studies have shown that consuming foods rich in fibre, and ω-3 PUFAs is associated with lower levels of pro-inflammatory biomarkers^(53,194–197)^. These findings support the notion that dietary interventions can reduce the risk of CVD by modulating inflammatory responses.

Studies evaluating the association between DII scores and CVD consistently report that consuming a diet with high pro-inflammatory potentials is associated with an increased risk of developing CVD^(193)^. A prospective cohort investigation conducted among 7216 men (aged 55–80 years) and women (aged 60–80 years) at high risk of CVD^(198)^ found that a higher DII score was significantly associated with an increased risk of CVD events, including myocardial infarction, stroke and cardiovascular death. Specifically, participants exposed to the highest quartile of the DII score had an adjusted hazard ratio (95 % CI) for CVD of 1·73 (1·15–2·60) after controlling for potential confounding variables^(198)^. According to Zhong *et al.*, individuals with higher levels of DII had increased risks of all-cause mortality (hazard ratio 1·22; 95 % CI: 1·06, 1·41) and cardiovascular mortality (relative risk 1·24; 95 % CI: 1·01, 1·51) compared to the lowest^(186)^. It was also suggested that a more pro-inflammatory diet was independently related to elevated risks of CVD and mortality in the general population^(186)^.

At the proximate level, oxidative stress, a state of imbalanced production of reactive oxygen species, free radicals and pro-oxidants, has been implicated in the development and progression of aforementioned cardiovascular events^(19)^. Considering the substantial impact of oxidative stress on these diseases, the emphasis on consuming antioxidant-rich foods, constituting a form of anti-inflammatory diet, has become a key focus for potential preventive strategies. The rationale behind this approach is rooted in the association between a diet abundant in fruits, vegetables, extra virgin olive oil, legumes, nuts, fish and poultry (with limited red meat consumption) and favourable factors such as a lower glycaemic index. These dietary choices are characterised by elevated levels of beneficial bioactive food components, including polyphenolic flavonoids, dietary fibre, carotenoids, antioxidants, ω-3 fatty acids and various essential vitamins and minerals^(199,200)^. Such a diet leads to a notable decrease in oxidative stress^(201)^. On the contrary, oxidative stress triggers the activation of pro-inflammatory signaling pathways, which in turn can contribute to the inflammation associated with the diseases^(19)^. This unifying theory provides a framework for comprehending the relationship between oxidative stress and inflammation in the development of chronic diseases, such as CVD, and may direct to the development of new therapeutic approaches.

It is important to mention that certain anti-inflammatory dietary regimens like the MD contain high levels of polyphenols, which are abundant antioxidants commonly derived from extra virgin olive oil, fruits, vegetables, seeds and nuts. Polyphenols have been recognised for their anti-inflammatory properties^(202)^, and the consumption of extra virgin olive oil, rich in polyphenols, has been linked to decreased risks of cardiovascular events (e.g. myocardial infarction and stroke) as well as reduced mortality rates^(203,204)^. In a sub-study of the PREDIMED trial examining the impact of polyphenol intake on inflammation and cardiovascular risk^(205)^, Medina-Remón *et al.* discovered that higher polyphenol intake from a MD was associated with significant reductions in inflammatory biomarkers related to atherosclerosis, namely VCAM-1, ICAM-1, IL-6, TNF-α and monocyte chemoattractant protein-1. Cardiovascular risk factors also showed improvements with decreases in systolic and diastolic blood pressures and an increase in HDL-cholesterol levels^(205)^.

In addition to aforementioned findings, the CORDIOPREV study demonstrated that long-term adherence to the MD was associated with decreased progression of atherosclerosis, as evidenced by the reductions in carotid intima-media thickness and plaque height, indicators of atherosclerosis progression, regardless of changes in lipid profiles and body weight^(206)^. Furthermore, the MD resulted in a lower incidence of major cardiovascular events, particularly among male participants, based on a study involving 1002 patients with established CHD, solidifying the clinical recommendation of the MD as an effective intervention for secondary cardiovascular prevention^(207)^. These effects are suggested to stem from the diet’s anti-inflammatory and antioxidant properties, which may attenuate atherosclerotic disease beyond conventional risk factor modifications. Such mechanisms have been underscored by the decreased levels of inflammatory biomarkers, like IL-6 and CRP, and improvements in endothelial function^(208)^, proposing an alternative pathway through which the MD may provide cardiovascular protection.

### Autoinflammatory diseases

Autoinflammatory conditions such as rheumatoid arthritis and psoriasis belong to a unique class of NCD that share an underlying factor in common with other conditions reviewed here: chronic inflammation^(209)^. While genetic and environmental factors contribute to the development of these diseases, there is growing evidence suggesting that nutrition can modulate immunological and inflammatory responses^(210)^. A comprehensive understanding of the interplay among nutrition, immunity and inflammation is therefore essential for the management of autoinflammatory NCD and has important implications for public health policy and clinical practice.

#### Rheumatoid arthritis

Rheumatoid arthritis (RA) is a persistent and complex condition that primarily affects the joints. It is an autoimmune disorder, meaning the body’s immune system mistakenly attacks healthy joint tissue, leading to inflammation and joint damage. Common symptoms of RA include joint pain, stiffness and swelling, as well as the potential for joint destruction and disability. Risk factors for developing RA include both genetic and non-genetic factors, such as smoking, diet, changes in the gut microbiome, gender and ethnicities^(211)^. Additionally, there is evidence to suggest that dietary factors may act as triggers for the development of RA in individuals who are genetically predisposed to the condition^(212)^.

When it comes to nutritional therapy for RA, the MD and fish consumption are among the most commonly cited dietary interventions^(212–216)^. The generic mechanisms by which dietary interventions influence RA parameters include the decrease in levels of inflammatory cytokines, regulation of oxidative stress and modification of the gut microbiota^(217)^. Some studies mentioned adjusting the balance of ω-6 to ω-3 fatty acids and boosting antioxidants to alleviate inflammation in RA^(213)^. A key aspect of this approach is reducing arachidonic acid, which has been found to be particularly beneficial. A low consumption of the ω-3 fatty acid eicosapentaenoic acid tends to increase the amount of arachidonic acid in cells, and eicosapentaenoic acid replaces arachidonic acid in cell membranes, which reduces arachidonic acid available for pro-inflammatory molecules production. Furthermore, eicosapentaenoic acid inhibits two groups of enzymes involved in eicosanoid biosynthesis, the cyclooxygenases and lipoxygenases, thereby limiting the production of inflammatory molecules^(218,219)^.

Moreover, ω-3 fatty acids have also been found to modulate the activities of macrophages, neutrophils, T cells and other immune cells^(220)^. They modulate inflammatory processes by being converted into bioactive lipid mediators such as resolvins and protectins. These mediators actively resolve inflammation by reducing the production and activity of pro-inflammatory cytokines like TNF-α, IL-1β and IL-6, which are critically involved in the inflammatory cascade of RA^(221,222)^. This results in less joint pain and stiffness as well as improved overall disease management^(223)^. Additionally, ω-3 fatty acids may inhibit the differentiation of CD4 + T cells into pro-inflammatory Th17 cells^(224)^. This modulation helps in restoring the balance between regulatory T cells (Tregs) and Th17 cells, which is often disrupted in RA patients.

Vegetables and fruits have been reported to have beneficial effects, possibly due to their antioxidant properties^(225,226)^. Additionally, the intake of fibre, prebiotics and polyphenols supports a diverse and balanced gut microbiota towards an anti-inflammatory profile^(227,228)^, whereas diets high in saturated fats, sugar and processed foods may disrupt its composition^(229)^. A key mechanism involves the production of metabolites by gut bacteria that modulate immune cell function. For instance, the short-chain fatty acid butyrate inhibits pro-inflammatory cytokine production and enhances the regulatory functions of T cells^(230)^. Moreover, the gut microbiota dysbiosis can contribute to increased intestinal permeability, allowing the translocation of bacteria and their components into the bloodstream and trigger inflammation^(231)^, which further activates immune cells, exacerbating RA conditions.

Some works have shown that individuals with arthritis tend to consume less nutrient-dense foods (i.e. fruits, vegetables, plant-based protein, whole grains, legumes and seafood), while consuming more empty energy, saturated fats and added sugars^(232)^. This finding is consistent with research that has shown a poor dietary quality in individuals with RA^(233–235)^. Therefore, future studies can focus on determining the optimal dietary approach considering the relationship between dietary factors, inflammation and immune response regulation.

#### Psoriasis

The aetiology of psoriasis is a multifaceted process, involving the dysregulation of both the innate and adaptive immune systems. The activation of T helper (Th) cells, specifically Th1 and Th17 cells, leads to an excessive production of pro-inflammatory cytokines, including interleukins (IL-1, IL-6, IL-23, IL-22, IL-17 and IL-33), TNF-α and IFN-γ^(236,237)^. This cytokine cascade plays a central role in the development of psoriasis by promoting hyperproliferation and angiogenesis, resulting in the characteristic skin lesions and articular involvement of psoriatic arthritis^(238)^. People diagnosed with psoriasis have a higher likelihood of developing obesity, diabetes, high cholesterol levels, CVD and inflammatory bowel diseases. They also often have a diet characterised by a higher intake of fatty foods and a lower intake of fibre-rich foods and fish^(239)^. Many studies have established a connection between psoriasis and chronic inflammation^(240)^. Given that inflammation can be regulated through diet, it is not surprising that research has focused on the effects of nutrition in the development and severity of psoriasis, as well as its impact on treatment responses.

Indeed, a diet abundant in anti-inflammatory nutrients can help alleviate the severity of psoriasis, and a low consumption of MUFA is the best dietary predictor of the Psoriasis Area and Severity Index score, an indicator of the disease’s clinical severity. MUFA may in fact reduce inflammation in individuals with psoriasis^(241)^. Some research has demonstrated low vitamin D levels may also contribute to the development of psoriasis by influencing the growth and maturation of skin cells called keratinocytes^(242)^. Other nutrients, such as short chain fatty acids, vitamin B_12_, Se, dietary fibre, genistein and probiotics, have all been found to alleviate psoriasis by suppressing inflammatory pathways or inducing regulatory T cells^(239)^. While most studies have focused on the individual nutritional components of the diet and their potential role in psoriasis, there is evidence to suggest that overall dietary patterns and lifestyle factors may also contribute to the disease’s pathogenesis.

A cross-sectional case–control study of 62 psoriasis patients examining the relationship between adherence to the MD and psoriasis severity found a significant association between the two^(243)^. This finding was further confirmed by Phan *et al.* ^(244)^, Caso *et al.* ^(245)^, Korovesi *et al.* ^(246)^ and Monlina-leyva *et al.* ^(247)^, which found that adherence to an anti-inflammatory MD was negatively correlated with psoriasis severity. Suggestions have also been made for the use of gluten-free diets, ketogenic diets, energy-restricted diets for obese patients and vegetarian diets in control of psoriasis^(239,248)^. However, although there is currently no widely accepted nutritional therapy for treating psoriasis, intakes of foods rich in antioxidants and with anti-inflammatory properties are highly recommended. Patients are advised to limit their consumption of red meat, saturated fatty acids, refined and added sugars, highly processed foods and alcohol. Instead, a diet rich in vegetables and fruits, which are good sources of antioxidants, as well as nuts, extra-virgin olive oil and marine fish, which provide healthy fats from the ω-3 family, should be prioritised. Whole-grain cereal products and legumes should also be included in their diet, and in some cases, patients may need to follow a gluten-free diet and consider supplements of vitamin D^(248)^.

### Cancer

As previously mentioned, obesity is frequently an underlying factor in chronic inflammation, as adipose tissue releases pro-inflammatory cytokines^(249)^, increases oxidative damage^(250)^ and disrupts gene transcription^(251)^. These elements are recognised contributors to cancer development. Moreover, several dietary factors are identified as contributors to cancer risk, particularly those associated with elevated levels of serum inflammatory markers such as IL-1β, -4, -6, -10, CRP and TNF-α^(252)^. The consumption of a pro-inflammatory diet can sustain inflammation over time, fostering the emergence of cancers in specific body regions^(252)^. Additionally, transcription factors linked to the inflammatory process can be activated by cytokines and other biomarkers, thereby facilitating the initiation and progression of cancer^(253)^.

The recognition of correlations between inflammatory conditions and an elevated risk of cancer in affected organs initially suggested the possibility that cancer might be a disease rooted in inflammation^(254)^. Immune cells, including macrophages, cytotoxic T cells, and natural killer cells, generate cytokines that can either support tumour immunity or contribute to the development of chronic inflammation^(255)^. However, the tumour microenvironment lacks normal immune protective mechanisms, resulting in a prevailing inflammatory state that fosters the growth and progression of cancer cells. Cytokines in this inflammatory milieu can enhance tumour cell growth, invasion, metastasis and angiogenesis, while concurrently impairing the function of immune cells^(255)^.

Furthermore, the NFκB pathway establishes a crucial link between inflammation and cancer development^(256)^. NFκB, a transcription factor, exhibits heightened activity in various cancer types and plays a pivotal role in the progression of these diseases^(257)^. During organogenesis and inflammatory responses, NFκB regulates genes essential for cell survival, proliferation, angiogenesis and invasion, crucial for organ growth and healing^(258)^. However, in cancers with activated NFκB, these genes promote cancer development and progression. Various carcinogens, inflammatory agents and factors in the tumour microenvironment can activate NFκB, leading to uncontrolled cell growth and tumour formation^(257)^. Inhibiting NFκB activation has proven effective in slowing down cancer growth and progression^(259–261)^. Hence, dietary components that suppress NFκB activity present promising avenues in cancer prevention and treatment. Examples include isoflavones, flavanols, stilbenes, curcumin, organosulfur compounds and ω-3 fatty acids^(254)^.

Consuming a balanced diet that includes fruits and vegetables, specifically cruciferous vegetables, garlic and other foods high in folic acid, vitamin B_12_, Se and vitamin D, may protect against cancer and reduce the risk of breast cancer, colorectal cancer and prostate cancer by 60–70 % and lung cancer by 40–50 %^(262)^. A diet rich in fibre-dense foods, such as whole grains, and moderate in dairy products may be associated with a reduced incidence of various types of cancer, including colorectal, lung, stomach, breast, colorectal, esophageal and oral cancer^(263–268)^. Conversely, animal products, particularly those cooked at high temperatures, may increase the risk of colorectal, stomach and prostate cancers^(269)^. These findings are consistent with the outcomes of dietary patterns rich in antioxidant and anti-inflammatory compounds, such as the MD, having the potential to reduce the incidence of cancer.

Another study investigating dietary inflammatory potential^(270)^ found that individuals in the highest category of the DII had a significantly elevated risk of colorectal cancer, with a relative risk of 1·40 (95 % CI: 1·26, 1·55; I^2^ = 69 %, *P* < 0·001), indicating a 40 % increased risk compared to the reference category. Additionally, there was a linear association between the DII score and CRC risk, with an increase of 1 point in the DII score corresponding to a 7 % increased risk of CRC^(270)^. These findings provide moderate evidence of heterogeneity. Wang *et al.* ^(271)^ also observed a significant correlation between the energy-adjusted DII (E-DII) and breast cancer mortality with a hazards ratio (HR) E-DII tertile (T) 3 *v*. T1 of 1·47 (95 % CI: 0·89, 2·43; *P*_trend_: 0·13) and multivariable-adjusted HR of 1·10 (95 % CI: 1·00, 1·22) for each-unit increment, suggesting an anti-inflammatory diet as a potential method for improving survival among breast cancer survivors.

### Cognitive health

Cognitive impairment is an escalating global concern, with projections estimating over 100 million adults diagnosed with dementia by 2050, presenting a substantial challenge to public health^(272)^. Extensive research indicates that chronic inflammation, as reflected by elevated levels of pro-inflammatory cytokines in the blood (termed peripheral inflammation), may adversely affect cognitive function and elevate the risk of dementia, particularly among older adults^(272)^. Peripheral inflammatory markers in the blood have been associated with neurodegeneration, suggesting increased neuroinflammation through neuronal and hormonal pathways^(273)^. Thus, systemic peripheral inflammation can adversely impact cognitive function and mental health by creating an inflammatory environment within the central nervous system. This, in turn, triggers the activation of microglia and astrocytes, adopting pro-inflammatory phenotypes that contribute to the progression of neurodegenerative processes^(274)^. Specifically, this can lead to excessive phosphorylation of tau protein, the aggregation of β-amyloid peptides into oligomers, activation of the complement system and neurotransmitter degradation, ultimately resulting in cognitive decline and dementia^(274)^.

Furthermore, inflammation has been closely linked to the development of Alzheimer’s disease^(7)^. The chronic activation of microglial macrophages in the brain, a natural aging process that accelerates under pathological conditions, results in an overproduction of pro-inflammatory cytokines such as IL-1β, IL-6 and TNF-α^(275,276)^. This production contributes to a detrimental cycle of neuroinflammatory processes involving amyloidosis, neuronal death, neurodegeneration, cortical thinning, reduced brain volume and occurrences related to cerebral vascular disease^(273)^.

As this paper has repeatedly shown, dietary elements are potent regulators of inflammation. Thus, it is not surprising that the relationship between diet and inflammation has been linked to a range of neurological disorders, including AD, Parkinson’s disease, multiple sclerosis, schizophrenia, bipolar disorder and depression^(277)^. For example, individuals who consume higher amounts of red and processed meat and fried foods and lower amounts of whole grains have been shown to have elevated levels of inflammatory markers (IL-6, TNF-α and CRP) and experienced more rapid cognitive decline as they aged^(278,279)^. Data show that consuming a diet rich in anti-inflammatory foods such as fruits, vegetables, legumes, nuts, herbs, and spices that are sources of polyphenols, ω-3 fatty acids and essential vitamins and minerals, while avoiding those that are pro-inflammatory, can create a beneficial environment for the brain and reduce the risk of neurological diseases^(277)^.

The MD and dietary approaches to stop hypertension (DASH) have garnered particular attention as potential methods for the prevention of dementia due to their well-established anti-inflammatory effects^(280–282)^, and both diets have demonstrated correlations with moderative cognitive decline^(283,284)^ and reduced risk of Alzheimer’s disease^(285–288)^. A greater alignment with the MD has been linked to improved cognitive function, slower cognitive decline and a lower risk of cognitive impairment and Alzheimer’s disease^(273)^. Observational studies have consistently shown that higher adherence to the MD is associated with more favourable brain structural and functional characteristics, which may protect against neurodegeneration, and increased cortical thickness, greater brain volumes, slower rate of hippocampal atrophy, improved structural connectivity and reduced accumulation of beta-amyloid (Aβ) in both mid-life and older age individuals^(273)^. The DASH diet, on the other hand, focuses on the consumption of fruits, vegetables, whole grains, poultry, fish, low-fat dairy products and nuts with low Na, fats, sugar and red meat intakes^(289)^. Moreover, alcohol is eliminated. This dietary approach has also demonstrated improved cognitive function and attenuated cognitive decline in older individuals with higher DASH scores^(283,289)^. However, these findings were inconsistent for both diets when they were tested in RCT or intervention studies^(273)^.

Despite conflicting results, both MD and the DASH diet have been associated with lower levels of inflammatory markers^(290)^. Consequently, further investigation is crucial to determine whether inflammation induced by specific diets can impact cognitive function and contribute to impairment. The Women’s Health Initiative study found that higher DII scores were linked to increased levels of inflammatory biomarkers, including IL-6, hs-CRP and TNF-α2^(291)^. Hayden *et al.* concluded that diets promoting inflammation may elevate the risk of developing mild cognitive impairment or dementia^(292)^. Moreover, a significant correlation exists between higher dietary inflammatory potentials, as measured by the DII, and an elevated risk of cognitive decline and earlier onset of cognitive impairment^(292)^. This was affirmed by Skoczek-Rubinska *et al.* in postmenopausal women, where the consumption of a diet with the highest pro-inflammatory profile significantly increased the risk of cognitive impairment compared with those adhering to the most anti-inflammatory diet^(293)^. A one-point increase in the continuous variable of energy intake-adjusted DII (E-DII) was associated with a significant rise in the odds of cognitive impairment, with an OR of 1·55 (95 % CI: 1·19, 2·02, *P* = 0·003)^(293)^. The HELIAD study also identified an underlying dose-response relationship between DII scores and the incidence of dementia^(294)^.

In 2015, Morris *et al.* introduced the Mediterranean-DASH diet intervention for neurodegenerative delay (MIND) diet, which focused on particular dietary components recognised for their neuroprotective properties and evaluated their effects on cognitive decline with the support of existing research on diet and dementia^(295)^. The MIND diet constitutes most of an anti-inflammatory dietary approach and is predominantly plant-based, emphasising the consumption of whole grains, leafy greens, fish, poultry, berries, nuts, beans, olive oil and wine in moderation. It simultaneously advocates for minimal consumption of animal and saturated fats such as red meats, butter, cheese and margarine^(295)^. A recent meta-analysis of cohort studies revealed that higher MIND diet scores were associated with improved global cognitive function, with each three-point increase in scores corresponding to a significant adjusted difference of 0·110 in global cognitive function z-score (95 % CI: 0·060, 0·159, *P*_trend_ < 0·001), approximately equivalent to a 1-year reduction in age^(296)^. Specific dietary components such as nuts, fish and tea intake were independently linked to enhanced cognitive function, while the consumption of fried foods showed negative associations^(296)^.

Regarding depression or anxiety, adopting a long-term anti-inflammatory dietary pattern may help prevent the development of these conditions, while a pro-inflammatory dietary pattern may increase the risk of experiencing depression or anxiety^(297,298)^. The nutritional composition of the diet also significantly influences the makeup of the gut microbiome. Microorganisms in the gastrointestinal tract metabolise dietary components into signaling molecules, neurotransmitter precursors or catalyse the synthesis of neurotransmitters through dietary metabolism^(299)^, which in turn can contribute to the development of metabolic changes and inflammation^(300)^. The makeup and functioning of the microbiota undergo changes in several psychiatric disorders, including depression, anxiety and schizophrenia^(277)^. Gut microbiota play a pivotal role in influencing the gut–brain axis^(300)^, a two-way communication network connecting CNS and the enteric nervous system. This modulation occurs through the production of metabolites like short-chain fatty acids, which can directly impact the CNS. Changes in the gut microbiota have been linked to alterations in neurotransmitter levels, including serotonin and dopamine, crucial for mood and behaviour regulation^(277)^. Studies have suggested that probiotics, live microorganisms with potential health benefits, may have beneficial effects on depression and anxiety symptoms, as well as cognitive function^(301–303)^. Consuming diets abundant in fresh fruits, nuts and seeds is positively associated with the overall *α*-diversity of the gut microbiome. Specifically, there is a noteworthy correlation with various taxa belonging to the Bacteroidetes phylum, including anti-inflammatory species such as *Faecalibacterium prausnitzii* and *Eubacterium rectale* and *Eubacterium biforme* ^(304)^. In contrast, a diet high in red and processed meat was associated with an increase in pro-inflammatory bacterial strains, including those related to *Ruminococcus gnavus* and *Collinsella* spp.^(304)^. These findings suggest that a plant-based diet may not only promote a more diverse and beneficial gut microbiota but also support cognitive function by reducing inflammation and modulating the gut-brain axis.

### Discussion and conclusion

**Fig. 2.**
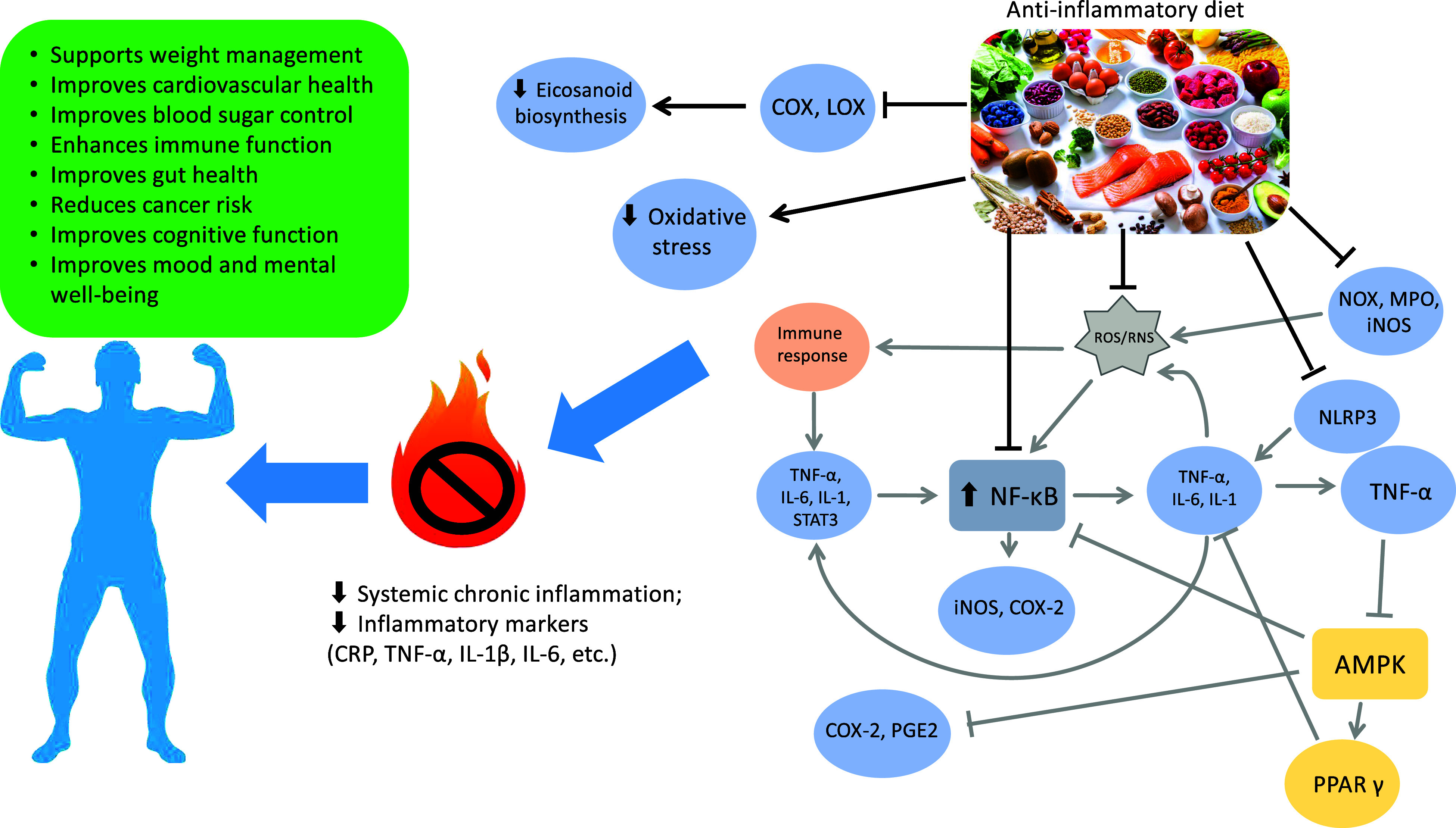
Effects of an anti-inflammatory diet: a visual overview. Gray arrows follow the pro-inflammatory pathway, black arrows and connectors denote the effects of an anti-inflammatory diet. COX, cyclooxygenase; LOX, lipoxygenase; ROS, reactive oxygen species; RNS, reactive nitrogen species; NADPH oxidase, NADPH oxidase; MPO, myeloperoxidase; iNOS, inducible nitric oxide synthase; NLRP3, nucleotide-binding domain, leucine-rich-containing family, pyrin domain-containing-3; NF-κB, nuclear factor-kappa B; STAT3, signal transducer and activator of transcription 3; COX-2, cyclooxygenase 2; PGE2, prostaglandin E2; AMPK, AMP-activated protein kinase; PPAR-γ, peroxisome proliferator-activated receptor γ; CRP, C-reactive protein; IL-1β, interleukin 1β.

The exploration of the impact of an anti-inflammatory diet on various health conditions underscores the importance of a holistic approach in managing chronic inflammation for the prevention and mitigation of non-communicable diseases. Tailoring the diet to an individual’s specific health needs can potentially influence the course of these conditions and contribute to overall health and well-being. The rise of anti-inflammatory diets is rooted in an enhanced understanding of the pro-inflammatory and anti-inflammatory properties of food, as demonstrated in Fig. 2, influenced by patterns like the MD. As research sheds light on the role of diet in modulating inflammation, these dietary approaches gain popularity.

To adopt an anti-inflammatory dietary pattern, it is recommended to replace refined grains with whole grains, substitute healthy fats (such as nuts, seeds and fatty fish) for saturated and trans fats and increase the consumption of colourful fruits and vegetables while reducing the intake of sugary and processed foods. Additionally, choosing lean protein sources like fish and legumes over red and processed meats, using herbs and spices (such as turmeric, ginger, garlic and cinnamon) in place of excessive salt and sugar and consuming probiotic-rich foods (such as yogurt and kefir) instead of processed and sugary snacks are advised. These dietary adjustments can help mitigate inflammation and promote overall health.

Moreover, adapting anti-inflammatory diets to different geographic locations and cultures involves incorporating the diversity of traditional diets that naturally align with anti-inflammatory principles. This approach respects cultural preferences and leverages local agricultural practices and seasonal food availability. For instance, the MD serves as a model for anti-inflammatory eating, with potential adaptations including local herbs and spices like saffron and za’atar^(305,306)^. South Asian diets can be enhanced by incorporating whole grains like barley, quinoa and black rice and promoting fish and plant-based proteins. Similarly, Sub-Saharan African diets can emphasise traditional leafy greens like moringa and amaranth^(307,308)^. East Asian diets can benefit from reducing refined grains and sugars while promoting traditional fermented foods like tempeh, kimchi and miso. Latin American diets can focus on plant-based foods, healthy cooking methods and reducing added sugars, while incorporating local herbs and spices like cumin, oregano, chili peppers and cilantro for their anti-inflammatory benefits.

Moving forward, more research is required to identify more comprehensive and integrated biomarkers to fully comprehend the intricate and multifaceted nature of chronic inflammation. While existing biomarkers such as CRP, IL-1β, IL-6 and TNF-α have been useful in demonstrating the relationship between inflammation and disease risk, they provide only limited mechanistic insights and do not capture the full range of anti-inflammatory pathways that may also play a role in inflammation-related disease prevention. Additional biomarkers, such as monocytes, B cells, CD8 + T cell subsets, natural killer cells and CD4 + T cell subsets, that exhibit considerable variation among individuals are suggested as helpful diagnostic additions to gain insight into mechanisms and underlying causes of inflammation^(142)^.

In conclusion, while the elements of an anti-inflammatory diet have typically been assessed individually, adopting the dietary plan as a whole may yield the greatest benefits. This study has delved into the role of individual foods or food groups in non-communicable diseases (NCD). Unlike isolated assessment of single foods or nutrients, dietary patterns consider potential interactions and collective effects of multiple dietary components, offering more comprehensive insights into an individual’s dietary habits. The concept of dietary inflammatory potential, notably measured by indices like the DII, has garnered significant attention. However, it is crucial to recognise that these indices do not directly represent a specific diet; rather, they serve as tools to quantify potential inflammatory effects. To ensure the validity and reliability of these findings, further research and replication in diverse populations are essential, ultimately refining recommendations for personalised dietary interventions in the prevention and management of chronic diseases.
